# Compensatory variances of drug-induced hepatitis B virus YMDD mutations

**DOI:** 10.1186/s40064-016-3003-x

**Published:** 2016-08-12

**Authors:** Ying Cai, Ning Wang, Xiaomei Wu, Kai Zheng, Yan Li

**Affiliations:** 1 Medical Research Center, Southwest Hospital, Third Military Medical University, Chongqing, 400038 China; 2 Department of Infectious Diseases, No. 324 Hospital of PLA, Chongqing, 400020 China; 3 Department of Microbiology, Third Military Medical University, Chongqing, 400038 China

**Keywords:** HBV, Drug resistance, YMDD, Mutation, Covariance, Evolution

## Abstract

Although the drug-induced mutations of HBV have been ever documented, the evolutionary mechanism is still obscure. To deeply reveal molecular characters of HBV evolution under the special condition, here we made a comprehensive investigation of the molecular variation of the 3432 wild-type sequences and 439 YMDD variants from HBV genotype A, B, C and D, and evaluated the co-variant patterns and the frequency distribution in the different YMDD mutation types and genotypes, by using the naïve Bayes classification algorithm and the complete induction method based on the comparative sequence analysis. The data showed different compensatory changes followed by the rtM204I/V. Although occurrence of the YMDD mutation itself was not related to the HBV genotypes, the subsequence co-variant patterns were related to the YMDD variant types and HBV genotypes. From the hierarchy view, we clarified that historical mutations, drug-induced mutation and compensatory variances, and displayed an inter-conditioned relationship of amino acid variances during multiple evolutionary processes. This study extends the understanding of the polymorphism and fitness of viral protein.

## Background

Hepatitis B virus (HBV) is one of ideal models to study on the viral mutation and evolution, because the rapid and imprecise replication produce a high rate of non-synonymous mutations, which are able to provide the virus much more chances to adapt the circumstances, on the contrary, functional constraint of the viral gene weeds out those lethal mutations, so the HBV genomes present not only diversity, but also relative conservation (Zhang et al. 2010).

With the accumulation of HBV genome sequences, there are eight natural genotypes (A–H) identified around the world (Miyakawa and Mizokami 2003; Schaefer 2007), among which the genotype A is mainly prevalent in Europe, the genotype B and C are prevalent in China and nearby regions and genotype D is mainly from Africa and middle east, etc. (Zeng et al. 2005; Kao et al. 2002; Wang et al. 2007; Schaefer 2007). In these years, wide application of the nucleotide analogue results in the HBV drug resistance. The typical YMDD motif variation (rtM204I/V) could be found in the reverse transcriptase (RNA-dependent DNA polymerase, RT) from all the genotypes, after the patients with HBV infection took a long-term treatment of the *lamivudine* (Malmstrom et al. 2007; Zhang et al. 2003; Moskovitz et al. 2005). These provoke us great interests to study the evolutionary selection of HBV under this specific condition.

Recently, more and more research works illustrate that YMDD variation is not a single mutation of rtM204I/V in the RT, but companied by compensatory mutation, such as rtL180M, etc. (Thai et al. 2012; Zoulim and Locarnini 2009). On the other hand, HBV genotype is not only predictive of clinical outcomes but has also been associated with response to anti-virus treatment (Lin and Kao 2011; Akuta and Kumada 2005). Meanwhile, the YMDD variant types might be different among the genotypes (Li et al. 2006). Nevertheless, many aspects about molecular variation of different YMDD mutations and HBV genotypes are still obscure, further studies are needed to deeply reveal these issues. Fortunately, there has been plenty of information about HBV drug resistance available in the public sequence database now, which brings great convenience to address the question.

Given these, here we, using bioinformatics techniques, attempt to make a comprehensive investigation of the molecular variation of the RT region among genotype A–D, in order to improve understanding of the evolutionary characters under the condition of HBV drug resistance.

## Methods

### Data preparation

The amino acid sequences of HBV polymerase with the genotype information were obtained from a knowledge database for hepatitis B virus, HBVdb (Hayer et al. 2013). There were totally 3871 sequences from genotype A–D, out of which, 439 YMDD mutations were harvested from different genotypes. Moreover, the polymerase sequences with sub-genotype information were got from NBCI protein database for the reference during the phylogeny tree drawing.

### Screen of the variant sites

After a multiple alignment by Muscle module of Mega software (6.0 version) (Tamura et al. 2011), the RT regions were extracted from each polymerase protein sequences by our in-house Perl program, at the same time, they were divided into different groups according to the genotype, variant type and wild type.

Because single mutations commonly occur in the biological sequence, no matter among variant or wild type groups there might exist different amino acids in one site. Thus, it is needed to distinguish the drug-induced compensatory mutations from random mutations, here we adopted the naïve Bayes classification algorithm to estimate the amino acids in each site, which mostly exist in the variant samples rather than wild samples, according to the following formula:

$$t_{map} = \mathop {\arg \hbox{max} }\limits_{t \in T} P(a|t)P(t)$$

where the maximum posterior (t_map_) represents the mostly type (variant or wild type) that the amino acid of each site belongs to. The probability of mutative rate is denoted by P(t), referring to the index from an epidemiological investigation (Hann et al. 2008). The probabilities of every amino acids from different types P(a) were independently calculated by above collected data. Given this, if the amino acid with more than 35 % composition rate had a *t*_*map*_ belonging to the variant type, the variant site was screened out. In addition, the genotyping sites could be also identified between different genotypes by this approach.

### Identification of co-variant patterns

To completely display the co-variant characters, we constructed a complete induction algorithm to series-connect the changed amino acids into a combined pattern, according to above variant sites. Given this, the patterns of each sequence were obtained by our in-house Perl program. The percentage of each pattern was calculated at the same time.

Meanwhile, a co-evolution analysis was carried out in order to figure out the co-variant features of the above variant sites by using CAPS 1.0 (Fares and McNally 2006). Due to the limitation of this algorithm and the novel requirement for the hardware, we just select some representative sequences from every group to run CAPS at a 0.001 alpha value in our Ubuntu Server with the memory of 16G. In addition, the Fastcov 1.03, a newly-developed bioinformatics tool  (Shen and Li 2016), was used for covariant analysis of the full data. The results were used to be the additional evidence along with the former co-variance pattern analysis.

### Chi square test

A Chi Square Test was used to measure a goodness of fit between an observed and expected distribution of quantity of YMDD variants among different genotypes, the functions of CHITEST and CHIINV (probability, freedom degree) were used to calculate the Chi Square test statistic in Excel sheet. The difference of observed and expected distribution was determined by the *P* value less than 0.05.

### Phylogenetic analysis

Phylogenetic analysis was performed with maximum likelihood approach for revealing the evolutionary relationships of the co-variant mutations to the different sub-genotypes, and an phylogeny tree was constructed and drawn as a radiation graphic by Mega 6.0 (Tamura et al. 2011).

## Results

### Different occurrences of YMDD variants among genotypes

Although several types of YMDD variants were previously reported, the most frequent types were the YIDD and YVDD (Pan et al. 2007; Ono-Nita et al. 1999). Table 1 showed that the occurrence rates of two types of mutations were significant different among A–D genotypes. By Chi square test, the occurrence rates of the YIDD type were generally higher than those of YVDD in genotype B, C and D, but on the contrary, the rate of YVDD type was significantly higher than that of YIDD in genotype A (I/V ratio = 0.1, *P* = 0.001). Based on the coden degeneracy principle, any mutation occurred at the third nucleotide of the triplet coden would change the M into I, and only if the mutation of A->G at the first one of the triplet coden could change the M into V, else into L or R, but the latter two are quite rare in fact. It seemed the YIDD mutation should occur naturally higher than the YVDD. Interestingly, the reverse feature of genotype A exactly presented a fact that the mutation was subject to the restriction of function or structure much more than the coden degeneracy principle.

**Table 1:** Distribution of different YMDD variants with different genotypes

	Wild	YIDD	YVDD	I/V ratio
Genotype A	596	10	132	0.1
Genotype B	963	35	13	2.7
Genotype C	1118	83	66	1.3
Genotype D	755	74	26	2.8

## Variant characters of different YMDD mutations

**Fig. 1:**
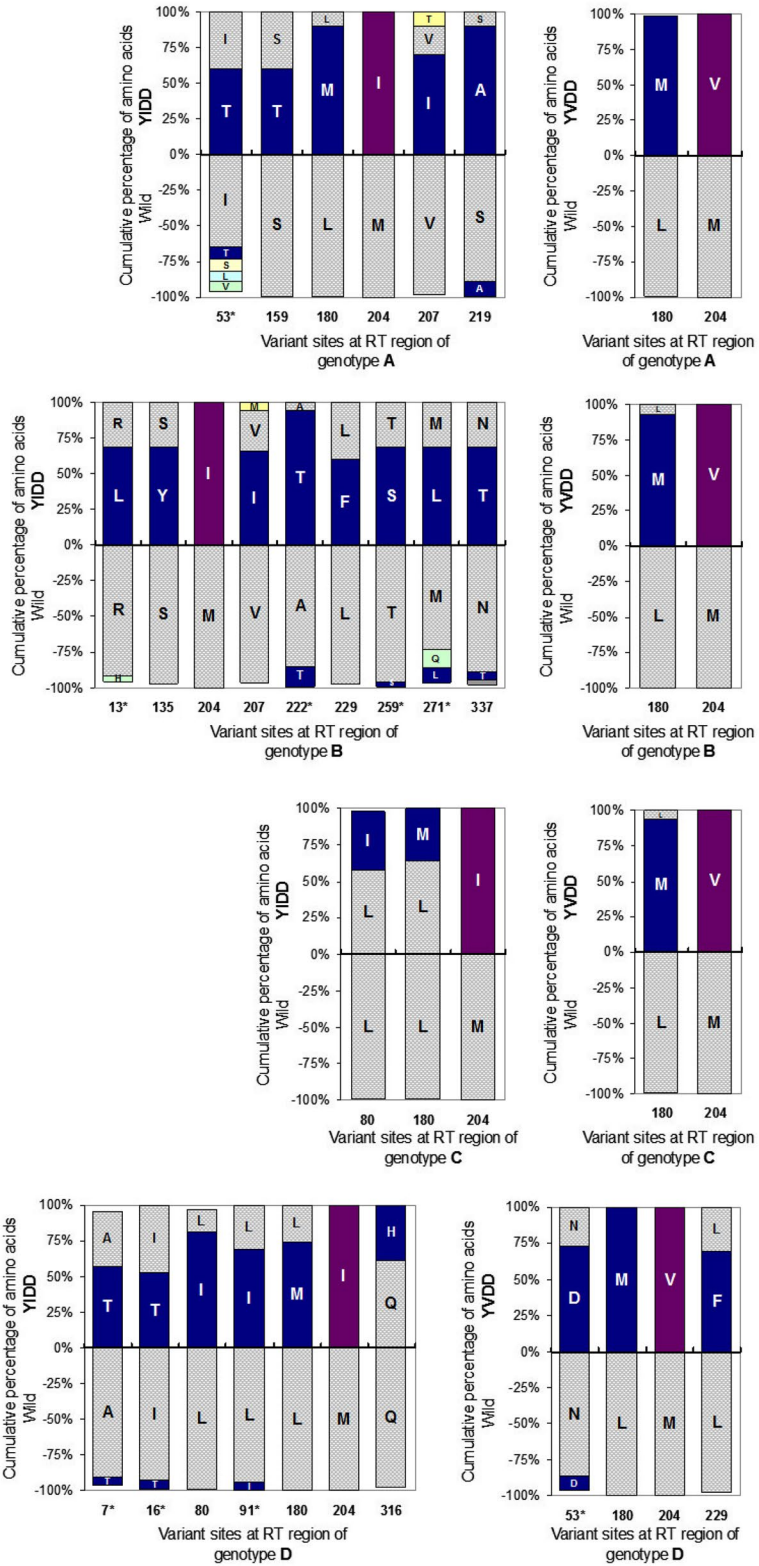
Amino acids composition at every variant site of the mutations in comparison to those of the wild types from different genotypes. *Note*: The minus Y axis is just used to display the wild type, which still means the positive value. The * represents the genotyping site

To investigate the variant character of the RT region, the comparative analysis was performed between the wild and the mutation groups from different genotypes, by the naïve Bayes classification algorithm. The variant sites where the amino acid composition were changed more than 35 % with a higher probability of mutation were harvested (Fig. 1).

Generally, the covariant sites and rates were not same in different mutational types and genotypes. The YIDD mutations (left column of Fig. 1) came along with much more variant sites than the YVDD mutations (right column of Fig. 1) in all the genotypes. Among these sites, the rtL180 M came along with rtM204I/V as previous reports, but here we noticed it was not along with rtM204I in genotype B and majority of genotype C. Meanwhile, more co-variances were detected at other sites.

Based on the large data of wild types, the single amino acid polymorphism could be found at many sites. While the covariance occurred at those sites, the amino acids composition ratio was generally inverted, such as those at site of 53 and 219, etc. It reflected the natural polymorphism provided the possibility for the selection. Interestingly, their converted ratios were always lower than that of rtM204I/V itself. So the non-synchronous conversion supported the fact of the compensatory variance after YMDD mutation.

Moreover, we noticed that some variances occurred at the historical genotyping sites (labeled by asterisk in Table 2), among which, new amino acid could appear, such as rtR13L in genotype B, but many of them trended to transform into other genotyping amino acid, such as rtL91I in genotype D, etc. Anyway, it displayed an interesting fact that the genotyping sites could be influenced once again while the much more variances took place under the new condition and pressure, and the single amino acid polymorphism reflected that these sites were less conserved, but the flexibility might be useful for the structural balance.

**Table 2:**
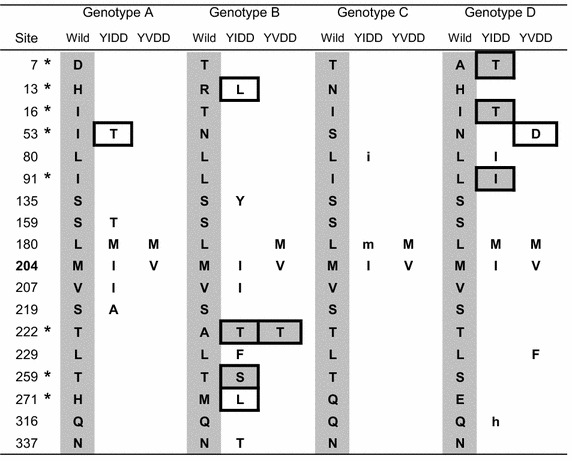
Changing features of every variant at different sites

The genotyping sites (the genotype-characteristic amino acid located) are labeled by asterisk, where the changed amino acids are rounded by a black frame and those transforming to other genotype are in gray background

## Co-variant patterns of YMDD variants

To clarify the covariance followed by the YMDD mutation, we investigated the multiple-sites combined co-variant status. Using the complete induction method to series-connect the variant sites, the co-variant patterns were checked out from every RT sequence.

Table 3 showed the co-variant patterns were commonly existed in all kinds of mutational types and genotypes. It demonstrated the previously reported rtL180 M was not the unique compensatory variance. Especially, the co-variant patterns in the YIDD type were much longer than those in the YVDD type. Meanwhile, the co-variant patterns were different in every genotype.

**Table 3:** Co-variant patterns of different YMDD variant types and genotypes

YIDD_A (53, 159, 180, 204, 207, 219)		YVDD_A (180,204)		YIDD_A (13,135,204,207,222,229,259,271,337)		YVDD_B (180,204)		YIDD_C (80,180,204)		YVDD_C (180,204)		YIDD_D (7,16,80,91,180,204,316)		YVDD_D (53,180,204,229)	
TTMIIA	40.0 %	MV	98.5 %	LYIITFSLT	57.1 %	MV	92.3 %	IMI	8.4 %	MV	93.9 %	–TIIMI–	29.7 %	DMVF	69.2 %
TTMIIA	10.0 %	–V	1.5 %	LYIIT–SLT	8.6 %	–V	7.7 %	VMI	1.2 %	–V	6.1 %	T–IIMIH	25.7 %	DMV–	3.8 %
T–MIIA	10.0 %			LYIMT–SLT	2.9 %			I–I	31.3 %			T–IIMI–	8.1 %	–MV–	26.9 %
–TMIIA	10.0 %			—I–TF—–	2.9 %			–MI	26.5 %			—I–MI–	4.1 %		
—MIIA	10.0 %			—I–T——	22.9 %			V–I	1.2 %			TT—–IH	12.2 %		
—MI–A	10.0 %			—IM——–	2.9 %			—I	31.3 %			TT—–I–	5.4 %		
—–I—	10.0 %			—I———	2.9 %							——–I–	1.4 %		

The letter represents the mutative amino acid, the symbol of “–” masks the unchanged site

Because the co-variant patterns expressed multiple status in every column (Table 3), it presented a dynamic transforming process. As the CAPS analysis, inter-molecular co-evolving sites were classified into different subgroups, for instance, the subgroup consisted by 53 and 204 covariant sites, and that by 207 and 219 covariant sites, etc., but the CAPS could not synchronously detect all of these pattern sites. Taken together, it reflected that the co-variance went through several steps during the evolutionary process, and the multiple-sites combined co-variance trended to an ideal state after rtM204I/V mutation. Moreover, the Fastcov obtained quite similar covariant patterns to those in Table 3 based on the intergroup data, but not from the whole data. That meant the drug-induced single mutation promoted different compensatory mutations related to the subgroups.

## The evolution of HBV followed with YMDD mutation

For the multiple-sites co-variances caused by drug-induced YMDD mutation, it was necessary to deeply figure out their evolutionary relationship. As we known, HBV genotypes differ by more than 8 % using the sequence of its complete genome or 4 % using the sequence of small surface protein (Schaefer 2005). Here using naïve Bayes classification model, we checked out that at least 5 % of RT region were different among genotype A–D, but the sub-genotypes differed by at least 4 amino acids. Therefore, the mutational RT sequences with 4 or more amino acids combined co-variant patterns were targeted for evolutionary analysis.

**Fig. 2:**
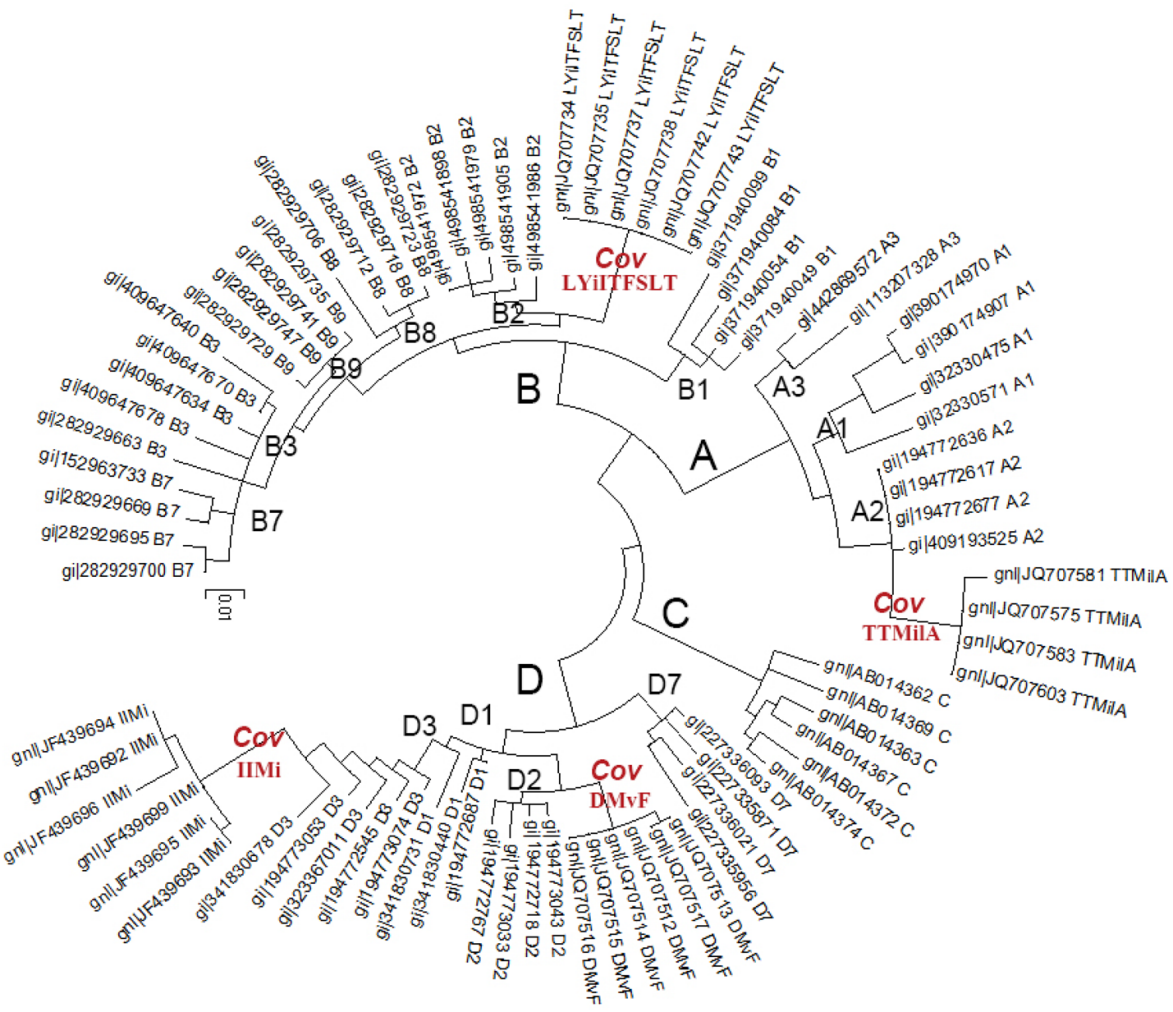
Phylogeny tree of the co-variant strains against the known sub-genotypes. *Note*: The co-variant amino acid at site of 204 was in lowercase

Based on the maximum likelihood algorithm, the phylogeny tree was drawn for the co-variant RT sequences against the NCBI recorded sub-genotypes (Fig. 2). It showed that the co-variant sequences still located in their original genotype, but all of them were clustered into independent branches (called Cov branches in Fig. 2) against the known sub-genotypes. The result suggested that these co-variances resulted in the formation of new sub-groups. It reflected that the flexibility of RT sequences provided the chance of adaptive selection, and drug-induced mutation could push the evolution of RT region.

## Discussion

In this study, we have made a comprehensive investigation on the YMDD variants from genotype A, B, C and D. By the naïve Bayes classification algorithm and the complete induction method based on the comparative sequence analysis, this study presents more detailed subsequent mutations followed with the *lamivudine*-induced rtM204I/V mutation. In contrast to the previous reports based on the longitudinal analysis (Thai et al. 2012; Zoulim and Locarnini 2009), this study conducts a cross-sectional analysis of integrity data by using bioinformatics techniques. From the hierarchy view, we clarify that historical mutations, drug-induced mutation and compensatory variances, and demonstrate the co-variant patterns associated with the YMDD variant types and HBV genotypes. This study displayed an inter-conditioned relationship of amino acid variances during multiple evolutionary processes.

For the *lamivudine*-induced mutation itself, the data shows that the genotype A tends to acquire rM204V mutation type, but the number of covariant sites of rM204V is much less that of rM204I. Because the historical genotyping mutations had already appeared before the drug-induced mutation, we could distinguish the subsequent amino acid changes as the compensatory variance. Although the *lamivudine*-induced rtM204I/V mutation might occur in any genotype, the compensatory mutations are not consistent in different genotypes. For instance, the previous studies reported a high incidence rate of rtL180M followed by YMDD variance, here we deeply figure out that the rtL180M is generally followed by rtM204V, but it is less followed by rtM204I in genotype C, and rare rtL180M by rtM204I in genotype B. Moreover, a set of subsequent amino acid changes took place after drug-induced mutation, and formed different co-variant patterns in different YMDD variant types and HBV genotypes. It reflects that compensatory variances of amino acids are subject to the former morphology of viral protein. That means that the RT is not only diversity, but also restricted. On the other hand, because some co-variant sites could influence the genotyping sites, this interesting adaptive selection under a special pressure suggests the flexibility of these historical sites take part in the structural balance as well.

Drug-induced mutation seems begin with one site (Song et al. 2012), and be followed with more or less compensatory mutations. Because the 3D structural modeling has shown that rt180 in the rt173–189 helix is close enough to interact with rtM204 of the YMDD loop (Allen et al. 1998), the rtL180M is ever regarded as the rational explanation to the compensatory mutation. But here we provide new evidence that rtL180M hardly exists in rtM204I of genotype B and C, it means the rtL180M is not a unique key site to remain 3D structural balance, and other covariance also could contribute to the structural and functional stability of the RT.

In the previous study, we have illustrated the different evolutionary selection of multiple genes from the longest overlapping region of HBV genome, and testified functional restriction based on the amino acid sequence finally determined the selection, no matter which codon site the mutation occurs in the nucleotide sequence. Different from the pressure in overlapping region, this study demonstrates the adaptive selection under drug pressure. But both of the results consistently support that the functional restriction and structural stability essentially determine the HBV selection.

The diversity of co-variant patterns reflects a powerful fitness of viral protein, and it puts up a serious challenge to the anti-HBV therapies. So this study supports that the drug combination, i.e. *lamivudine* and *adefovir* combination therapy, might be useful to increase the difficulty of evolutionary selection. In spite of this, the multiple-drug resistance might still occur (Inoue et al. 2011; Chotiyaputta and Lok 2009; Mello et al. 2012; Yatsuji et al. 2007). Fortunately, some drugs, i.e. *tenofovir* or *entecavir*, have rarely been reported to be resistant (Agarwal et al. 2015; Colonno et al. 2006). It suggests that some crucial sites in amino acid sequence should be unchangeable where any mutation might be lethal for the virus.

## Conclusions

From the hierarchy view, we clarified that historical mutations, drug-induced mutation and compensatory variances. Although occurrence of the YMDD mutation itself was not related to the HBV genotypes, the subsequence co-variant patterns are different, which are related to the YMDD variant types and HBV genotypes. This study not only extends our understanding of molecular evolution and powerful fitness of viral protein, but also a subtle relationship of structural polymorphs and the function, which should be considered for anti-virus drug design, development and treatment.
